# Feasibility and placement accuracy of a sensor-guided intraosseous device with an automated stop in a small-bone rat cadaver model: a single-arm pilot study

**DOI:** 10.1016/j.resplu.2026.101500

**Published:** 2026-09-21

**Authors:** Markus Ortner, Clemens Hacker, Alexandra Kaider, Johann Stanek, David Russ, Ewald Unger, Victor Schmidbauer, Gunpreet Coudert Oberoi

**Affiliations:** a Centre for Medical Physics and Biomedical Engineering, Medical University of Vienna, Vienna, Austria; b SVAN Technologies GmbH, 8010 Graz, Austria; c Preclinical Imaging Lab (PIL), Department of Biomedical Imaging and Image-Guided Therapy, Medical University of Vienna, Vienna, Austria; d Institute of Clinical Biometrics, Center for Medical Data Science, Medical University of Vienna, Vienna, Austria; e Department of Biomedical Imaging and Image-guided Therapy, Medical University of Vienna, Austria

**Keywords:** Infusions, Intraosseous, Infant, Newborn, Emergency treatment

## Abstract

**Background:**

Rapid intraosseous (IO) access is critical in emergency care when venous access is not feasible. IO access is particularly prone to error in small animals and human neonates with narrow medullary cavities. This pilot study evaluated the feasibility and placement accuracy of a novel sensor-guided IO drilling device with an automatic stop mechanism in a rat cadaver model.

**Methods:**

Seven thawed Sprague-Dawley rat cadavers underwent 14 tibial insertions by a single experienced operator using a standardised protocol. Placement was assessed by computed tomography (CT), with successful insertion defined as intramedullary positioning of the needle tip. Two blinded readers independently classified each insertion. The principal outcome was the proportion of successful placements; a prespecified device-evaluable analysis excluding user-related targeting errors was exploratory**.**

**Results:**

Nine of 14 insertions were correctly positioned, yielding a success rate of 64.3% (9/14; 95% CI 35.1–87.2%). In the device-evaluable analysis, success was 81.8% (9/11; 95% CI 48.2–97.7%). Three lateral displacements and two overpenetrations occurred. Reader agreement was complete (*κ* = 1.00). The medullary cavity narrowed from 6.02 mm at the proximal metaphysis to 3.13 mm at the diaphysis, with cortical thickness of 0.54–0.57 mm; proximal epiphyseal outer diameter of 8.07 mm. No device malfunction was identified.

**Conclusion:**

In this small-bone cadaver model, the automated-stop intraosseous device achieved CT-verified intramedullary placement in most attempts and functioned as intended. As this single-arm pilot lacked a comparator and CT assessed needle-tip position rather than functional access, the findings are limited to placement accuracy and are hypothesis-generating.

## Highlights

• In small-bone cadavers, 9 of 14 sensor-guided intraossoeus insertions were intramedullary on CT (64.3%, 95% CI 35.1–87.2%).
• Automated stop activated after cortical breach as intended; overpenetrations were distal, where the medullary cavity narrows.
• CT confirms needle-tip position but not functional intraossoeus access, so the findings describe placement accuracy only.
• This single-arm, single-operator pilot is hypothesis-generating and does not establish superiority over existing devices.
The results support further preclinical validation and device refinement for potential veterinary and neonatal applications.

## Introduction

Birth asphyxia remains a leading cause of neonatal death, accounting for approximately one million deaths worldwide each year, an estimated 30% of which could be prevented by effective resuscitation at birth.^1^, ^2^, ^3^, ^4^ Fewer than 1% of all newborns require advanced cardiopulmonary resuscitation in the delivery room,^5^, ^6^ rising to 6–10% among preterm (<32 weeks' gestation) and very-low-birth-weight infants who may need chest compressions with or without epinephrine.^7^, ^8^, ^9^, ^10^ Advanced resuscitation depends on the rapid delivery of drugs and fluids, and therefore on immediate, reliable vascular access, precisely the step that is hardest to achieve in the smallest patients. Intraosseous (IO) access is the preferred route in neonates when umbilical venous cannulation (UVC) is delayed or not feasible.^4^, ^11^ Essentially, any drug or fluid that can be given intravenously (IV) can be given by the IO route, and IO access can often be established faster than IV access under emergency conditions.^12^ In both veterinary and human emergency care, IO access is therefore a valuable route when time and venous collapse work against the operator, as bone acts as a non-collapsible vein.^13^

Despite these advantages, IO access remains underused due to lack of training, age- and weight-specific equipment and awareness and hence is prone to error in the smallest bones. Most available systems, manual, spring-loaded, or powered, were developed for older children and adults and are not intended for use under 3 kg body weight, where thin cortices, narrow medullary cavities, and reduced metaphyseal mineralisation leave a very small margin for error. This elevates the risks of malposition and over-penetration.^14^, ^15^, ^16^ Because the neonatal medullary cavity may be only 1.5–3 mm across, a needle that advances a few millimetres too far breaches the posterior cortex, while one angled incorrectly exits the bone entirely; both cause extravasation of drugs and fluids into surrounding soft tissue. Documented serious complications of IO access include compartment syndrome^17^^,^^18^ tibial fracture, osteomyelitis, growth-plate injury, fat or bone embolism and popliteal artery damage^19^ and though rare, limb ischaemia^20^ and amputation^17^ have followed malposition^21^, ^22^ or posterior cortical penetration under high-pressure infusion. Reported first-attempt success is correspondingly low, pooled at 54.1% in clinical and 44.1% in cadaveric neonatal studies,^23^ far below the 80–95% typical in older children and adults. Crucially, in conventional powered systems drilling continues until the operator releases the trigger, so depth control rests entirely on tactile perception and reaction time, a margin that is easily overshot in a thin, poorly mineralised cortex where the loss-of-resistance signal is subtle. Current practice mitigates this only through fixed needle lengths, landmarking, and manual monitoring rather than real-time sensing, and no powered device dimensioned for bones of this calibre exists, leaving a clear unmet need for a size-adapted system that stops automatically at the medullary boundary.^24^

The novel safe-stop intraosseous access device (Generation 1) was designed to fill this gap. It incorporates a sensor-triggered automatic stop into a powered IO handpiece, ensuring that drilling halts after the cortex is breached. This feature intends to reduce overshoot and improve first-attempt medullary placement accuracy. Before live-animal testing, regulatory-grade device development requires preclinical verification of core performance, including functional reliability (such as auto-stop activation) and precise intramedullary placement.

This report presents a pilot cadaver study on rats aimed at (i) quantifying the rate of accurate intramedullary placement, (ii) verifying functional components, including the auto-stop mechanism. The rat tibia serves as an anatomically relevant, small-diameter long-bone model for preliminary validation of safety and performance parameters prior to live animal and clinical investigations. Because no FDA- or CE-cleared powered intraosseous access device is available for neonates or animals below 3 kg body weight, and because the smallest needle supplied with the dominant powered system (15 G, 1.8 mm outer diameter, 5 mm needle tip) approaches the outer diameter of the rat tibial diaphysis measured in this study, no comparator device could meaningfully be used in this model. The study was therefore designed as a single-arm feasibility verification, with performance interpreted descriptively and against published literature rather than tested for superiority.

## Methods

### Study design, specimens and ethics

This preclinical, cadaveric, single-arm pilot feasibility study was carried out at the GLP-compliant Centre for Biomedical Research and Translational Surgery at the Medical University of Vienna. This study was conducted using thawed rats (Sprague Dawley (SD), male, age: 8–12 weeks) obtained post-euthanasia. Only male animals were available, because the cadavers were obtained from prior experimental studies in which only male animals had been used; the implications are addressed in the Limitations. In accordance with Austrian law, the Federal Act on Animal Experiments, BGBl. I Nr. 114/2012 and institutional guidelines of the Medical University of Vienna, studies conducted exclusively on cadaveric animal material do not require ethics committee approval. All cadavers were handled in accordance with institutional biosafety protocols. The animals had been used in prior experimental studies and were euthanized upon reaching predefined humane endpoints in accordance with institutional guidelines. In line with the principles of the 3Rs (Replacement, Reduction, Refinement), cadaveric specimens were utilized to avoid the need for additional animal sacrifice specifically for the purposes of this study. The study was prepared with reference to the ARRIVE 2.0 guidelines, where applicable to a cadaveric animal study.

The device performance was interpreted against a single pre-specified literature benchmark: the pooled cadaveric first-attempt success rate of 44.1% for neonatal tibiae.^23^ This preclinical study was carried out in accordance with a predefined internal study protocol established before the start of the study. The protocol outlined the study procedures, primary endpoint, and exclusion criteria. Specimens were excluded if the tibia exhibited fractures, bone damage, or pre-existing bone disease that could influence device performance or assessment.

Seven (*N* = 7) thawed Sprague Dawley rat cadavers with no bone damage or pre-existing bone conditions were randomly selected by an independent individual not involved in the experimental procedure. Tests were performed on both the right and left hind limbs (*N* = 14). The chosen penetration site was the proximal tibia, located below the knee joint. The device operator has previous experience in bone drilling, is familiar with the device's functions and procedures, and has carried out all tasks.

### Device and sensing mechanism

As shown in Fig. 1**,** the novel intraosseous drilling device (a) consisted of a powered drill unit with a Quincke-type needle (Spinocan G22 × 3½ (B. Braun, Spinocan Germany) covered in capsule (b). The device features an automatic stop sensor that detects when the bone marrow cavity is reached and stops drilling immediately. Additional safety features ensure that drilling only occurs when the correct force is applied to the bone, and it cannot be reactivated once drilling has stopped. The handheld design is optimised for a pinch grip, enabling precise positioning and controlled creation of fine drill holes. The needle can be extended from the capsule to a length chosen by the user to improve skin penetration.

**Fig. 1:**
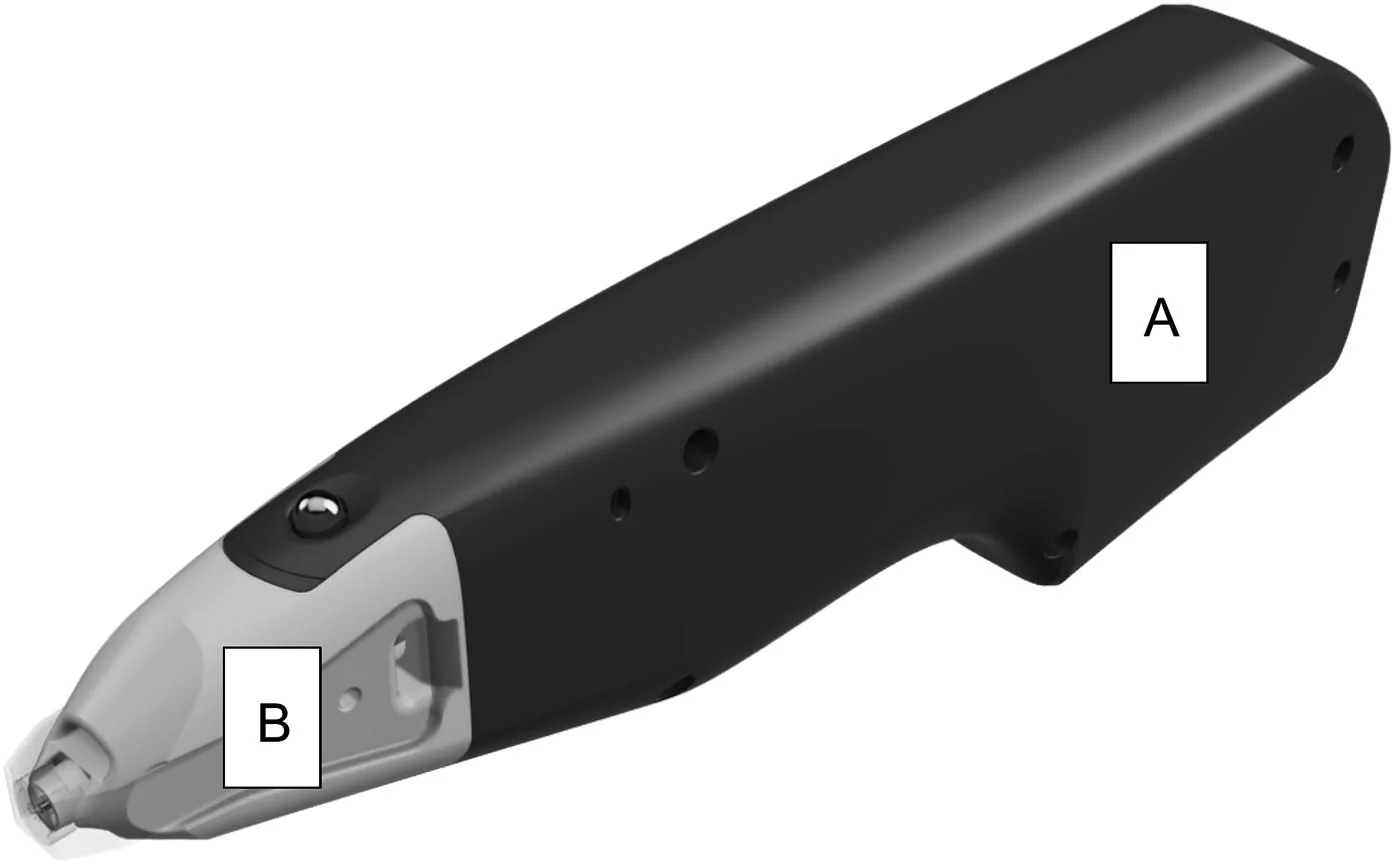
**Sensor-guided intraosseous device with an automated stop Generation 1**. *The device consists of a capsule (B, light grey) housing the needle, and a powered drill unit (A, dark grey) containing the integrated sensor mechanism*.

The device incorporates a patent-protected electro-magnetic auto-stop. A sensing element integrated into the drive shaft continuously transduces the mechanical resistance that bone offers to advancement of the needle. The mechanical resistance encountered at the needle tip is measured and transduced electro-magnetically; it is not electrical impedance or electrical resistance, not drive torque, and not an absolute axial force compared against a fixed threshold. Dense cortical bone presents high resistance to advancement, and at the moment the tip passes through the cortex into the medullary cavity that resistance collapses abruptly. It is this abrupt change, the transition itself rather than any absolute value, that the device detects and treats as the breakthrough signature, and the drive is de-energised within milliseconds of it. Because the trigger is a change rather than a magnitude, the principle is in this respect independent of needle-tip geometry and of the absolute forces involved, which is what allows it to operate at the sub-millimetre cortical thickness characteristic of small bones, where depth-limiting technologies calibrated to absolute torque or force in adult bone do not transfer.

Three properties distinguish cortical breach from other variations in resistance during drilling. First, the device responds to the rate at which resistance collapses rather than to its level, so the gradual variation produced by differences in bone density, needle sharpness or applied hand force does not trigger it. Second, the periodic component introduced by rotation of the drive is excluded from the signal used for detection. Third, drilling is enabled only while the sensing element remains loaded against bone, so loss of bone contact stops the drive rather than allowing it to continue, and the device cannot be run in air. Once the drive has stopped it cannot be reactivated without a full device reset, preventing inadvertent further advancement. The underlying principle is described in granted European patent EP 4 615 343; the sensing implementation, the signal-conditioning arrangement and the firmware parameters are proprietary and are not reported here.

A single device was used throughout for consistency. Fifteen needles (one spare) were loaded by an experienced engineer, who inspected the device for malfunction after each drilling and reloading cycle.

### Insertion procedure

All insertions were performed by a single operator experienced in bone drilling and familiar with the device, minimising variation in technique and judgment. Each insertion followed a standardised sequence: capsule preparation, anatomical palpation of the proximal tibia, automated drilling into the marrow cavity, and detachment of the drill followed by removal of the inner stylet, leaving the needle fixed in the bone.

### CT assessment, blinding and reader agreement

Cadavers were refrozen and later thawed for CT evaluation (Mediso nanoScan 122S PET/CT, Centre for Preclinical Imaging, Medical University of Vienna). Both hind limbs were dissected and scanned, yielding 14 datasets, each reviewed by an experienced operator to optimise image quality.

Placement was verified on CT (Mimics 24.0, Materialise, Belgium) by two image-analysis investigators independently, blinded to insertion sequence, procedural outcome, and each other. Each reader assigned every insertion to one of three predefined categories. Needles recorded by a reader as lying within cortical bone without intramedullary access were mapped to the lateral-displacement category, consistent with the pre-specified definition. Agreement between the two independent classifications before consensus is reported as the observed proportion of agreement and Cohen's *κ*. No discrepancy was found between the two readers. Successful placement was defined as a needle tip within the medullary cavity. Failures were classified a priori, before imaging, as device-related overpenetration (beyond the medullary cavity) or user-related lateral misplacement (needle medial or lateral to the cavity without intramedullary access), not adjusted post hoc.

### Anatomical measurements

Anatomical measurements were performed in seven rats. For each animal, either the left or right tibia was randomly selected for analysis. Measurements were taken at the proximal epiphysis, proximal metaphysis and diaphysis. The quantity measured differs between levels and is reported accordingly. At the proximal epiphysis, where no medullary cavity is present, the widest outer diameter of the bone was recorded. At the proximal metaphysis, the diaphysis and a fourth, more distal shaft level the widest internal diameter of the medullary cavity was recorded, measured between the endosteal surfaces of the opposing cortices, together with the cortical thickness at the same level. The distal level was included because both overpenetrations in this study occurred at insertions placed distal to the intended site, and the dimensions available to the needle there are the relevant ones for interpreting that failure mode. Outer diameter at these three levels is therefore not measured directly and is reported as a derived quantity, calculated as the medullary diameter plus twice the cortical thickness. Because an outer and an internal dimension are not comparable, the two are presented on separate rows of Table 2 and the epiphyseal value is not read as part of a proximal-to-distal series. Per-animal tibial measurements are displayed in the supplementary table.

**Table 1:** Independent and consensus CT classification of all 14 intraosseous insertion attempts.

**Animal**	**Side**	**Reader 1**	**Reader 2**	**Consensus classification**
Rat 1	Right	Lateral displacement	Lateral displacement	Lateral displacement
Rat 1	Left	Success	Success	Success
Rat 2	Right	Success	Success	Success
Rat 2	Left	Success	Success	Success
Rat 3	Right	Overpenetration	Overpenetration	Overpenetration
Rat 3	Left	Success	Success	Success
Rat 4	Right	Lateral displacement	Lateral displacement	Lateral displacement
Rat 4	Left	Overpenetration	Overpenetration	Overpenetration
Rat 5	Right	Success	Success	Success
Rat 5	Left	Success	Success	Success
Rat 6	Right	Lateral displacement	Lateral displacement	Lateral displacement
Rat 6	Left	Success	Success	Success
Rat 7	Right	Success	Success	Success
Rat 7	Left	Success	Success	Success

Total		9/3/2	9/3/2	9 success, 3 lateral, 2 overpenetration

Each insertion was classified independently by two readers blinded to insertion sequence, to procedural outcome and to each other, into one of three categories fixed before any imaging was reviewed: successful intramedullary placement, lateral displacement, or overpenetration. The two sets of classifications agreed on all 14 insertions (observed agreement 100%, Cohen's *κ* = 1.00), so the pre-specified consensus procedure was not required. Totals are given as success/lateral displacement/overpenetration. Where a reader recorded a needle as lying within cortical bone without intramedullary access, that record was mapped to the pre-specified lateral-displacement category.

**Table 2:** Tibial dimensions in rat cadavers (*n* = 7):

	**Prox. epiphysis**	**Prox. metaphysis**	**Diaphysis**	**Distal shaft**
Outer tibial diameter (mm)	8.07 ± 0.35	n/a	n/a	n/a
Medullary cavity diameter (mm)	n/a	6.02 ± 1.13	3.13 ± 0.47	1.22 ± 0.29
Cortical thickness (mm)	n/a	0.54 ± 0.12	0.57 ± 0.08	0.54 ± 0.07
Derived outer diameter (mm)	n/a	7.10 ± 1.15	4.27 ± 0.46	2.31 ± 0.35

One randomly selected tibia per animal (*n* = 7); data are mean ± standard deviation. The quantity measured differs between levels. At the proximal epiphysis, where no medullary cavity is present, the widest outer diameter of the bone was recorded. At the proximal metaphysis, the diaphysis and a distal shaft level, the widest internal diameter of the medullary cavity was recorded between the endosteal surfaces of the opposing cortices, together with cortical thickness at the same level. An outer and an internal dimension are not comparable and are therefore reported on separate rows. Derived outer diameter is calculated as medullary cavity diameter plus twice the cortical thickness and is not a direct measurement. For reference, the outer diameter of the Spinocan G22 needle used throughout is 0.70 mm.

### Statistical analysis

The sample size was determined a priori using an exact one-sample binomial test (*H*_0_: *p* = 50%, derived from pooled first-attempt success of powered devices in neonates^23^; *H*_1_: *p* = 90%, from internal bench testing; two-sided *α* = 0.05), requiring 12 evaluable tibiae for 0.89 power; 14 were enrolled to allow for exclusions.

The principal reported outcome is the all-inclusive analysis: the proportion of CT-verified intramedullary placements among all 14 insertions, counting lateral displacements as failures irrespective of their attributed cause. The pre-specified device-evaluable analysis, which excluded the three insertions classified a priori, before imaging, as user-related targeting errors, is retained and reported as a secondary exploratory analysis. Both estimates are reported throughout, and neither is presented in isolation.

Consistent with the exploratory pilot design, results are presented as point estimates with exact Clopper-Pearson 95% confidence intervals. Formal significance testing against the pre-specified null proportion, using the exact one sample binomial test, is reported for completeness only. Although the study was powered to detect a difference compared to a reference success rate, *p* values are only interpreted as exploratory due to the pilot study design. Overpenetration and user-error rates are reported as proportions of all insertions with Clopper-Pearson intervals. Anatomical measurements (one randomly selected tibia per animal, *n* = 7) are summarised as mean ± SD. Each tibia was analysed as an independent insertion attempt, consistent with the per-leg analytic convention used in the neonatal intraosseous access literature^23^; the potential for within-animal correlation between paired tibiae is acknowledged in the Limitations. Analyses were descriptive; given the pilot design, no adjustment for multiplicity was applied.

## Results

A representative successful placement is shown in Fig. 2. The left panel demonstrates the needle positioned within the rat tibia, while the right panel shows the corresponding CT image confirming successful intraosseous placement of the needle in the tibial bone.

**Fig. 2:**
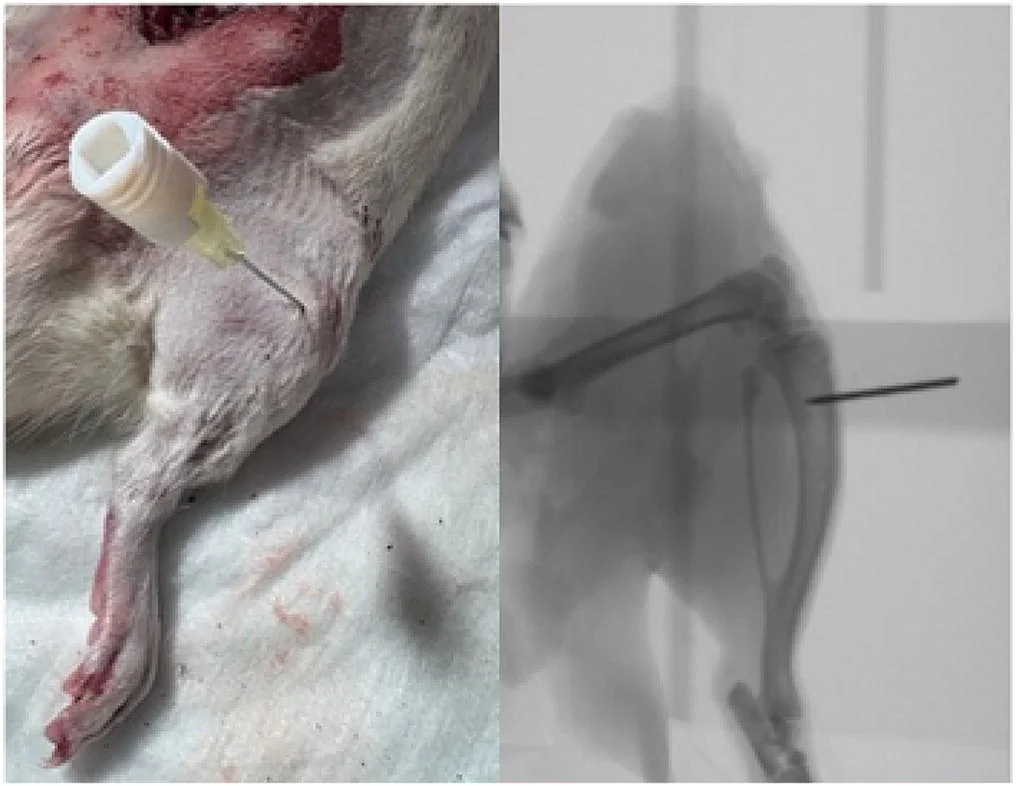
**Left: Photograph of the needle inserted into the rat tibia. Right: Corresponding CT image demonstrating correct positioning of the needle tip within the medullary cavity, representing a successful insertion attempt**.

Table 1 presents the final consensus classification for each individual tibial insertion according to animal and side. Of the 14 insertions, nine were classified as successful intramedullary placements, three as lateral displacements, and two as overpenetrations. Fig. 4 The two readers' independent classifications, recorded before any comparison between them, agreed on all 14 insertions (observed agreement 100%; Cohen's *κ* = 1.00). No discrepancy arose, so the pre-specified consensus procedure was not required. Because there was no discordance the confidence interval for *κ* is not estimable, and in a sample of 14 insertions across three categories the statistic should be interpreted with corresponding caution.

### Placement accuracy

#### Principal (all-inclusive) analysis

Counting all 14 insertions and scoring only intramedullary placement as success, the success rate was 64.3% (9/14; 95% CI 35.1–87.2%). This confidence interval includes both the pre-specified null proportion of 50% (exact binomial *p* value = 0.42) and the 44.1% pooled cadaveric benchmark^23^; the data are therefore compatible with a success rate that does not differ from either reference value.

#### Secondary device-evaluable analysis

After exclusion of the three insertions classified a priori as user-related lateral displacements, 11 insertions remained evaluable, of which nine were correctly positioned: 81.8% (9/11; 95% CI 48.2–97.7%; exact binomial *p* value = 0.065 testing against the null hypothesis of 50%). This interval likewise includes the pre-specified null. The analysis is exploratory and, because it removes failures from the denominator, is expected to be biased upward relative to clinical performance.

#### Failure modes

Among all 14 insertions, three lateral displacements corresponded to a user-error rate of 21.4% (95% CI 4.7–50.8%) and two overpenetrations to an overpenetration rate of 14.3% (95% CI 1.8–42.8%). Both overpenetrations occurred at insertions placed distal to the intended proximal tibial site. No device malfunction was identified on inspection after any drilling and reloading cycle.

### Bone diameter assessment

Bone diameter was assessed on CT images at predefined anatomical levels of the rat tibia, as shown in Fig. 3.

**Fig. 3:**
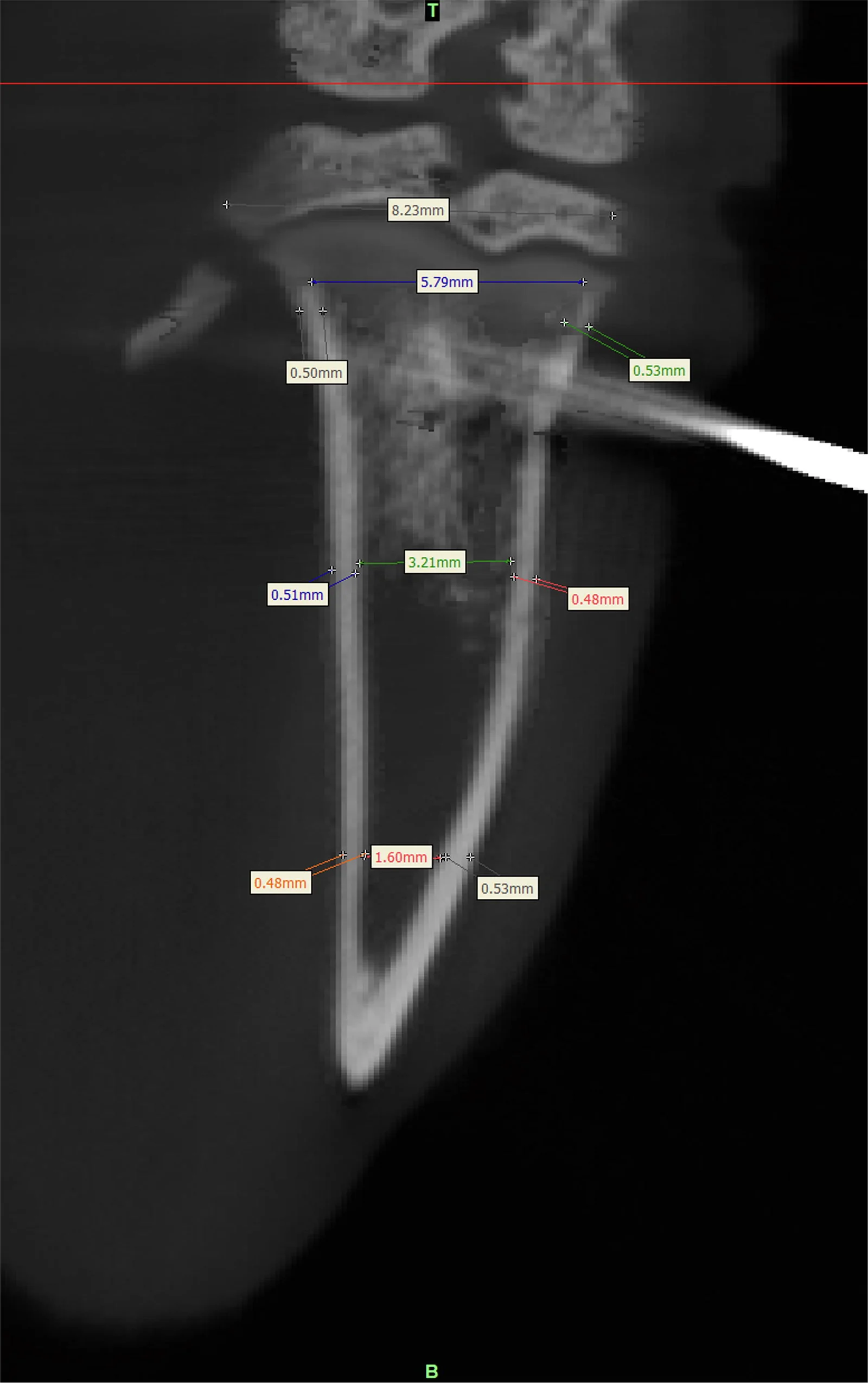
**CT image of the rat tibia showing the anatomical measurements.** *The outer diameter was recorded at the proximal epiphysis; the medullary cavity diameter and the cortical thickness were recorded at the proximal metaphysis and at the diaphysis. The widest dimension of the relevant structure was recorded at each location*.

**Fig. 4:**
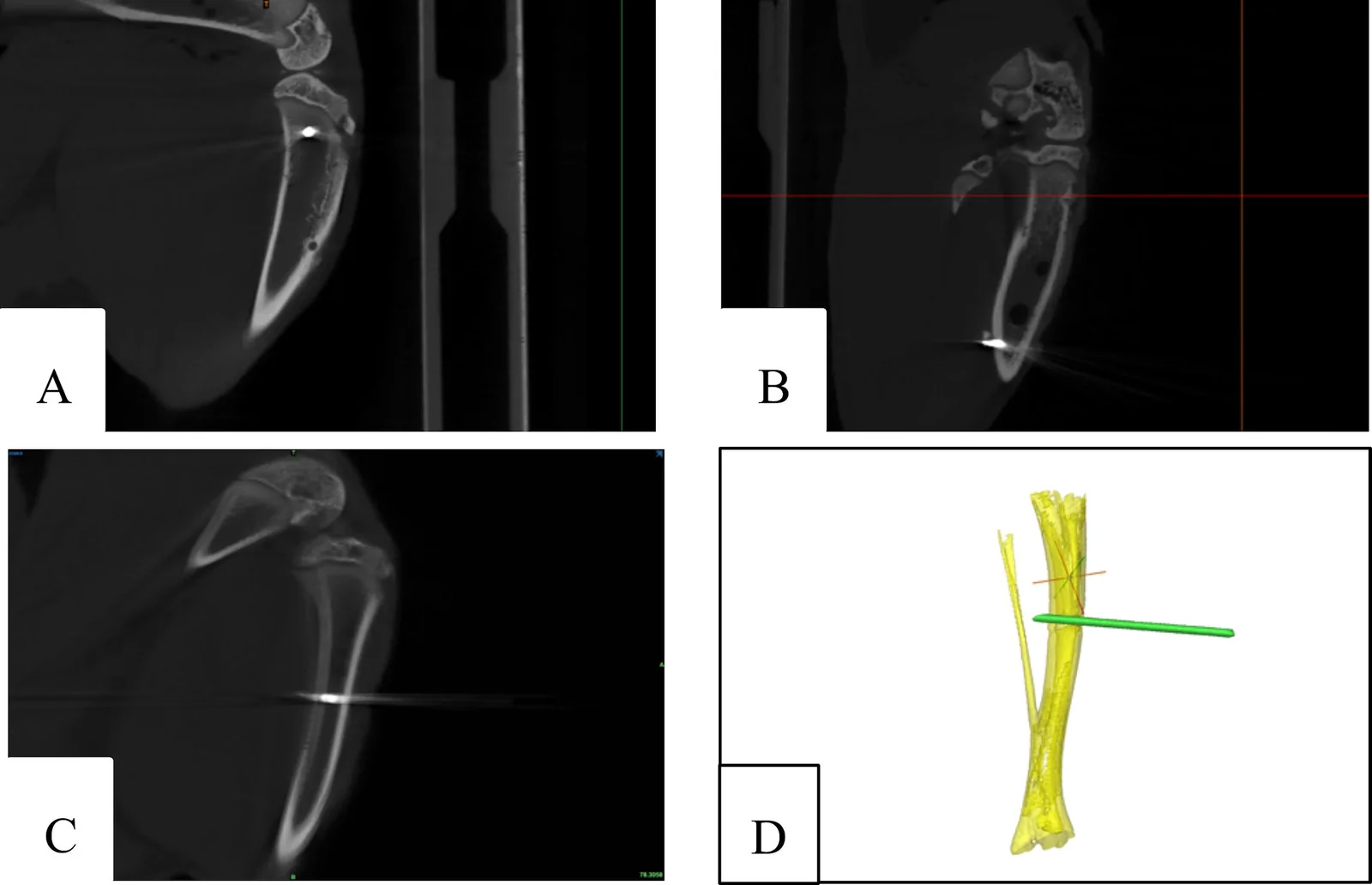
**Representative CT appearances of the three placement categories: (A) successful intramedullary placement, (B) lateral displacement with the needle outside the medullary cavity, (C) overpenetration with the needle tip beyond the far cortex, and (D) 3D reconstruction of overpenetration**.

The outer diameter of the proximal epiphysis was 8.07 ± 0.35 mm. The medullary cavity narrowed distally, from 6.02 ± 1.13 mm at the proximal metaphysis to 3.13 ± 0.47 mm at the diaphysis. At the fourth, more distal shaft level the cavity narrowed further, to 1.22 ± 0.29 mm, with a cortical thickness of 0.54 ± 0.07 mm and a derived outer diameter of approximately 2.31 mm. Cortical thickness was relatively consistent across regions, at 0.54 ± 0.12 mm at the proximal metaphysis and 0.57 ± 0.08 mm at the diaphysis, giving derived outer diameters of approximately 7.10 mm and 4.27 mm at these two levels respectively. For comparison, the outer diameter of the Spinocan G22 needle used throughout is 0.7 mm.

## Discussion

This study evaluated the feasibility and placement accuracy of a sensor-guided intraosseous (IO) device in a small-bone cadaver model, with particular attention to its automated stop mechanism. CT-verified intramedullary placement was achieved in 9 of 14 insertions, and the resistance-based motor cut-off activated as intended. Because the study was single-arm and included no comparator, and because CT establishes needle-tip position but not functional access, these findings describe placement accuracy in this model. They do not demonstrate that the device improves intraosseous access, reduces complications, or increases procedural reliability relative to existing devices.

### Interpretation of the two analyses

Two analyses of the same data are reported because they answer different questions and carry different biases. The all-inclusive analysis (64.3%) treats every insertion as it would present clinically: a needle lying lateral to the medullary cavity is a failed IO insertion regardless of whether the proximate cause was device behaviour or operator targeting, and end users cannot make that distinction at the bedside. It is therefore the clinically meaningful estimate and is reported as the principal outcome. The device-evaluable analysis (81.8%) isolates the sensing and stopping mechanism from targeting error, which is the relevant question for engineering verification, but it achieves this by removing failed attempts from the denominator and is consequently biased upward as an estimate of clinical performance. The failure taxonomy was fixed before any images were reviewed and was applied by readers blinded to procedural outcome, so the classification itself was not influenced by the result; nevertheless, attributing a lateral displacement to the operator rather than the device rests on the judgement that the device provides no lateral guidance, and reasonable readers may weigh that differently. Reporting both estimates, with the more conservative one as the principal result, is the approach least likely to overstate performance.

### Mechanistic and practical interpretation

The main innovation is rapid auto-stop at the bone marrow boundary. In conventional powered IO systems, drilling continues until the operator releases the trigger, placing full responsibility for depth control on tactile perception and reaction time.^24^, ^25^ In miniature bones, this operator-dependent stop mechanism carries a significant risk of overpenetration, as the medullary margin for error may be as narrow as 3 mm in neonates.^25^, ^26^ In this cadaver model, overpenetration was observed in 14.3% of all insertions. Direct comparison with prior figures is not possible because published rates differ in the denominator, device, and model where reported overpenetration was 29.3% of device failures in a neonatal simulation manikin,^25^ a proportion that is not comparable to our overpenetration rate among all insertions. Rather than a quantitative benchmark, these data indicate that overpenetration remained infrequent and, notably, was confined to insertions placed too distally, where the medullary cavity narrows.

By coupling a resistance-sensing signal to the motor cut-off, the Safe-Stop mechanism is *designed* to reduce dependence on operator reaction time and tactile feedback. Whether it achieves that reduction cannot be determined from a single-arm study: no comparator device was tested, so the observed overpenetration rate cannot be attributed to the auto-stop specifically, and a controlled head-to-head comparison is required to quantify any protective effect. Evaluation of the CT scans showed that overpenetration occurred distal to the optimal tibial puncture site. In this region, the rat tibia becomes progressively narrower and the medullary cavity correspondingly decreases in size, the medullary cavity measuring 3.13 ± 0.47 mm at the diaphysis and 1.22 ± 0.29 mm at the more distal level assessed, against a needle outer diameter of 0.7 mm, leaving little or no space for the needle to enter the bone marrow cavity. Consequently, the likelihood of overpenetration may increase when the device is positioned too distally. The risk of such events could be reduced by careful palpation and identification of the optimal insertion site. This was particularly challenging because no precise anatomical puncture location for this experimental setting was identified in the available literature, and the procedure was therefore conducted in an exploratory manner. Despite the suboptimal distal placement of the device, the auto-stop function performed as intended and triggered as soon as the needle penetrated the cortex, thereby potentially limiting further penetration and reducing the risk of adjacent tissue damage.

### Absence of a comparator

The absence of a comparator is the principal limitation of the design, and it constrains what can be concluded. No CE-marked or FDA-cleared powered intraosseous device carries an indication below 3 kg body weight, and the smallest needle supplied with the dominant powered system is 15 G (1.8 mm outer diameter, 5 mm tip length). That needle is supplied on 15 mm, 25 mm and 45 mm sets. In this study the rat tibia had a derived outer diameter of approximately 7.10 mm at the proximal metaphysis, so even the shortest 15 mm needle is more than twice the full width of the bone at the intended insertion level, and its 1.8 mm diameter is roughly a third of the 6.02 ± 1.13 mm metaphyseal medullary cavity it would have to sit within. A device of those dimensions cannot be arrested within this cavity by any fixed-length design, and the expected result of every attempt would be traversal of the bone or fracture rather than an interpretable placement. A head-to-head comparison in this model would therefore have been uninformative rather than merely unfavourable. This explains the design; it does not soften its inferential limits. Without a control arm it is not possible to determine whether the sensor-guided mechanism improves performance over existing devices, and superiority cannot be inferred from these data. A controlled comparison in a model that accommodates both devices, either human neonatal cadaveric bone or a validated bone surrogate of appropriate dimensions, is required to quantify any protective effect of the automatic stop and is planned as part of the confirmatory programme.

### Context within prior evidence and anatomical comparison

Published clinical and cadaveric work in infants and neonates documents lower first-attempt success and substantial malposition rates with adult-oriented devices, consistent with the anatomical mismatch between device dimensions and miniature bones.^23^ While direct cross-species comparison is not the aim of a pilot, our results in a small-bone model support the premise that sensor-guided stopping can mitigate size-driven risks and is therefore relevant for both veterinary small-animal emergencies and neonatal translation.

The rat tibia was anatomically smaller than the human neonatal tibia. The proximal metaphyseal medullary cavity measured 6.02 ± 1.13 mm with a cortical thickness of 0.54 ± 0.12 mm, giving a derived external diameter of approximately 7.10 mm, smaller than the 9.1 ± 1.6 mm reported in preterm stillborns of 25–27 weeks' gestation.^27^ That published value is an external proximal tibial metaphyseal diameter, so it is compared here against our derived external diameter at the same anatomical level rather than against the medullary measurement; the comparison is therefore like for like in both dimension and site. Reported neonatal medullary diameters vary widely with measurement site and maturity, from 4.0 mm in stillborn infants^28^ to 4.7 mm at the widest proximal level but only 2.1 mm at the actual insertion site.^29^ Hence, the rat proximal cavity is broadly comparable to published values, while its cortex (0.54 mm vs ∼0.9 mm) at the neonatal puncture site^24^ is thinner. A thinner cortex makes cortical breakthrough and the associated loss of resistance less noticeable, increasing overpenetration risk. The rat tibia therefore represents a technically demanding surrogate for the narrowest neonatal geometry.^27^

### Performance data

Interpreted against the most closely matched benchmark, which is the pooled cadaveric first-attempt success of 44.1% (95% CI 32.9–56.1%) for adult-derived devices in neonatal cadavers,^23^ defined identically as CT-confirmed intramedullary tip position on a per-leg basis, the present all-inclusive rate of 64.3% (95% CI 35.1–87.2%) and device-evaluable rate of 81.8% (95% CI 48.2–97.7%) are numerically higher. The confidence intervals overlap the benchmark, and the benchmark value itself lies within the interval of the principal all-inclusive estimate. This pilot therefore cannot establish a statistically significant or clinically meaningful improvement; the comparison is descriptive and hypothesis-generating. Both estimates share the same imaging endpoint and per-leg denominator as the benchmark, which keeps the comparison fair, but a numerical difference of this kind in 14 observations is fully compatible with chance. The overpenetration rate (14.3%) is comparable in magnitude to the contrast-leakage rates of 15.8%, rising to 41.7% in very-low-birth-weight specimens, reported in cadaveric neonatal work, though endpoints differ and no direct comparison is warranted.^29^ The user-error rate (21.4%) was roughly half the >40% attributed to positioning and technique in the literature.^30^ Overpenetration clustered at distal placements, where the margin for error narrows, underscoring that accurate site identification and operator training remain essential even with device safeguards.

### Independent validation and reproducibility

Several authors are employees or shareholders of the company developing the device, and one is a co-inventor of the underlying patent; all such interests are declared in full. This degree of investigator involvement is usual at this stage of device development, but it increases rather than removes the need for independent confirmation. Two features of the present study limit, without eliminating, that concern: CT adjudication was performed by two readers blinded to procedural outcome and to each other, with agreement reported before consensus, and the failure taxonomy was fixed before any imaging was reviewed. Neither addresses independence of conduct. The planned confirmatory investigation is designed accordingly: a prospective multicentre human post-mortem study across four European centres, with insertions performed by two independently trained physicians per subject under randomised operator-to-leg allocation, imaging adjudicated by independent radiologists under a blinded dual-reader model with third-reader consensus for discordant reads, and source-data verification by an independent contract research organisation. Reproduction of these findings by investigators with no role in device development remains necessary before any clinical claim is made.

### Device refinement following this study

The verification findings of this study were entered into the design-control record and have been addressed in subsequent design iterations. Two changes bear directly on the failure modes observed here. First, the needle capsule and its attachment-detection function, which were the source of an unreliable-attachment hazard, have been eliminated by design rather than controlled. Second, the current configuration replaces the earlier drive arrangement with a linear drive specified for neonatal and paediatric use, where the consequences of overpenetration are most severe, and adds explicit operator feedback: an indicator confirming that the automatic stop is armed before drilling begins, and a second indicator confirming that the stop has executed. Because both overpenetrations in this study occurred at insertions placed distal to the intended site, the site-identification problem is also being addressed procedurally: the protocol for the confirmatory investigation restricts insertion to the ultrasound-defined optimal proximal tibial site, with anatomical diagrams and step-by-step instructions in the instructions for use, site identification covered in investigator training, and confirmation of the site recorded on a procedural checklist. Overpenetration remains a pre-specified secondary safety endpoint in that study.

### Limitations

This exploratory, single-arm, single-operator, single-device cadaver study has several constraints.

First, it was not powered as a confirmatory trial. Only 11 of the planned 12 evaluable tibiae remained after exclusions, reducing power to approximately 0.70, and the confidence interval around every estimate reported here is correspondingly wide.

Second, there was no comparator, for the reasons set out under absence of a comparator. Superiority over existing devices cannot be inferred, and the observed overpenetration rate cannot be attributed to the automatic stop in the absence of a control arm.

Third, the study has not been independently validated. The device was developed by several of the authors and the study was conducted within the developing institutions. Reproduction by investigators with no role in device development is required before the findings can be regarded as established.

Fourth, all insertions were performed by a single experienced operator familiar with the device. A stated purpose of the device is to reduce operator dependence, and that purpose cannot be tested with one operator. Inter-operator variability was not assessed, and performance in the hands of operators with different levels of experience, including those encountering the device for the first time, is unknown. This is a substantial limitation on generalisability and on any claim of reduced operator dependence.

Fifth, success was defined by CT needle-tip position. Cadaveric imaging cannot demonstrate functional intraosseous access (aspiration, infusion patency, flow rate, leakage, extravasation or pressure characteristics), nor the physiological consequences of overpenetration. All statements in this report are therefore limited to placement accuracy.

Sixth, only male animals were used, because the cadavers were obtained from prior studies in which only male animals were available. Sex-related differences in bone density, cortical thickness and geometry are documented in rodents, and their effect on the resistance signal on which the automatic stop depends was not assessed.

Seventh, a single device and a spinal needle (Spinocan G22) used off-label limit generalisability. The absence of standardised rat tibial anatomical landmarks contributed to distal misplacement and is likely to have contributed to the lateral displacements; metal artefact from the needle may also affect CT delineation of the tip.

Eighth, specimens underwent repeated freeze–thaw cycles, which can alter cortical mechanical properties and therefore the resistance signal on which the device's cut-off depends; performance in fresh or living, perfused bone may differ.

Finally, both tibiae of each animal were analysed as independent insertions, following the per-leg convention standard in this field.^23^ Because paired limbs from the same animal may be correlated, the effective sample size may be smaller than the nominal 14, and the confidence intervals should be interpreted accordingly.

#### Implications and next steps

The results support conducting GLP live-animal studies to assess physiological safety, including infusion patency, extravasation, and histopathology, as well as confirmatory testing on human cadavers to establish depth margins at clinically recommended landmarks. They also indicate three specific requirements for the next stage: a comparator arm in a model that accommodates both devices; evaluation by multiple operators of differing experience, so that operator dependence can be measured rather than assumed; and independent conduct and adjudication. Because cadaveric CT confirms tip position but not functional access, future work should incorporate contrast-enhanced CT or micro-CT as a confirmation modality.

## Conclusion

In this small-bone cadaver model, the automated-stop intraosseous device achieved CT-verified intramedullary placement in 9 of 14 insertions (64.3%; 95% CI 35.1–87.2%), and its auto-stop function activated as intended on cortical breach. As a single-arm, single-operator feasibility study without a comparator, and one in which functional intraosseous access was not assessed, these results describe placement accuracy only. They are hypothesis-generating rather than confirmatory and do not establish that the device performs better than existing intraosseous devices. They support progression to controlled GLP live-animal studies (with infusion patency, extravasation and histopathology endpoints) and to independently conducted human-cadaver work establishing depth margins at clinically recommended landmarks, alongside continued design refinement for veterinary and neonatal applications.

## Patents

The device under development builds on the patent EP4615343B1, An apparatus for penetrating through an anatomical structure, for which Ewald Unger is listed as a co‑inventor; this patent is related to penetration/IO‑type instrumentation. The patent is owned by the Medical University of Vienna.

## CRediT authorship contribution statement

**Markus Ortner:** Writing – review & editing, Writing – original draft, Visualization, Validation, Project administration, Methodology, Investigation, Formal analysis, Data curation, Conceptualization. **Clemens Hacker:** Writing – review & editing, Software. **Alexandra Kaider:** Writing – review & editing, Formal analysis, Data curation. **Johann Stanek:** Writing – review & editing, Data curation. **David Russ:** Writing – review & editing, Software. **Ewald Unger:** Writing – review & editing, Investigation. **Victor Schmidbauer:** Writing – review & editing, Validation, Data curation. **Gunpreet Coudert Oberoi:** Writing – review & editing, Supervision, Methodology, Investigation, Funding acquisition, Conceptualization.

## Funding

This work was funded by the Austrian Research Promotion Agency (FFG) through the Spin-off Fellowship Program (Grant No. 909600) and by Austria Wirtschaftsservice Gesellschaft mbH (aws) through the Deep Tech Preseed Grant (P2507205-PSL01). The funding bodies had no involvement in the study design, data collection, analysis or interpretation, manuscript preparation, or the decision to submit the manuscript for publication. The corresponding author had full access to all study data and held final responsibility for the decision to submit.

## Data availability statement

The datasets generated and analysed during the current study are available from the corresponding author upon reasonable request.

## Declaration of competing interest

G.C.O. is the Chief Executive Officer and a shareholder of SVAN Technologies GmbH, the company developing the device evaluated in this study. E.U. is a shareholder of SVAN Technologies GmbH and a co-inventor of the patent underlying the device (EP4615343B1). M.O., E.U., and D.R. are employees of SVAN Technologies GmbH. C.H., E.U. and D.R. contributed to device engineering as part of their work at the Medical University of Vienna. G.C.O., E.U., and M.O. also hold part-time appointments at the Medical University of Vienna. The device evaluated in this study is under active commercial development by SVAN Technologies GmbH. The remaining authors declare no competing interests.

## Appendix A Supplementary material

Supplementary Data 1 Supplementary table 1: Anatomical measurements of the tibia of each animal.
