# Supplementary material for: Feasibility and placement accuracy of a sensor-guided intraosseous device with an automated stop in a small-bone rat cadaver model: a single-arm pilot study
Source: Resusc Plus. 2026 Sep 21;32:101500. doi: 10.1016/j.resplu.2026.101500 (PMC13659475; doi:10.1016/j.resplu.2026.101500)
Supplement: Supplementary file 1 [file mmc1.docx]

**Supplementary Table**

| Animal | Epiphysis outer diameter | Metaphysis medullary | Metaphysis cortex | Diaphysis medullary | Diaphysis cortex | Distal shaft medullary | Distal shaft cortex | Metaphysis derived outer | Diaphysis derived outer | Distal shaft derived outer |
| --- | --- | --- | --- | --- | --- | --- | --- | --- | --- | --- |
| Rat 1 | 7,87 | 4,37 | 0,42 | 2,95 | 0,68 | 1,58 | 0,65 | 5,21 | 4,31 | 2,88 |
| Rat 2 | *7,55* | *4,96* | 0,60 | *2,59* | 0,60 | *1,09* | *0,60* | 6,16 | 3,79 | 2,29 |
| Rat 3 | 8,23 | 5,79 | 0,52 | 3,21 | 0,50 | 1,60 | 0,51 | 6,83 | 4,21 | 2,62 |
| Rat 4 | 8,51 | 5,95 | 0,72 | 3,10 | 0,65 | *0,87* | *0,49* | 7,39 | 4,40 | 1,85 |
| Rat 5 | 7,90 | 6,37 | 0,54 | 3,23 | 0,50 | 1,13 | *0,47* | 7,45 | 4,23 | 2,07 |
| Rat 6 | 8,46 | 7,40 | 0,61 | 4,06 | 0,55 | 0,97 | 0,58 | 8,62 | 5,16 | 2,13 |
| Rat 7 | 7,97 | 7,31 | 0,37 | 2,77 | 0,50 | 1,32 | *0,50* | 8.05 | 3,77 | 2,32 |
| Mean (all 7) | 8,07 | 6,02 | 0,54 | 3,13 | 0,57 | 1,22 | 0,54 | 7.10 | 4,27 | 2,31 |
| SD (all 7) | 0,35 | 1,13 | 0,12 | 0,47 | 0,08 | 0,29 | 0,07 | 1,15 | 0,46 | 0,35 |
